# Long Non-Coding RNA PNKY Modulates the Development of Choroidal Neovascularization

**DOI:** 10.3389/fcell.2022.836031

**Published:** 2022-02-21

**Authors:** Lianjun Shi, Xue Han, Chang Liu, Xiumiao Li, Shuting Lu, Qin Jiang, Jin Yao

**Affiliations:** ^1^ The Affiliated Eye Hospital, Nanjing Medical University, Nanjing, China; ^2^ The Fourth School of Clinical Medicine, Nanjing Medical University, Nanjing, China; ^3^ Eye Institute, Eye and ENT Hospital, Shanghai Medical College, Fudan University, Shanghai, China; ^4^ NHC Key Laboratory of Myopia Fudan University, Key Laboratory of Myopia, Chinese Academy of Medical Sciences, Shanghai, China; ^5^ Shanghai Key Laboratory of Visual Impairment and Restoration, Fudan University, Shanghai, China

**Keywords:** long non-coding RNA, choroidal neovascularization, polypyrimidine bundle binding protein 1, miRNA, PNKY

## Abstract

Long non-coding RNAs (lncRNAs) have been widely implicated in human diseases. Our aim was to explore the regulatory role of changes in the expression levels of PNKY and its linked signaling networks in mediating stress-induced choroidal neovascularization. PNKY expression levels were reduced in mice by laser and exposure of endothelial cell to hypoxic stress. PNKY silencing exacerbated the formation of CNV in a laser-induced CNV model and an *ex vivo* model, while overexpression inhibited CNV development. Silencing or overexpression of PNKY altered the viability, proliferation, migration, and tube-forming capacity of endothelial cells *in vitro*. Mechanistically, through the lncRNA–RNA binding protein–miRNA interaction analysis involving loss of function and gain-of-function experiments, we found that lncRNA PNKY inhibited the binding of miR124 to PTBP1 and maintained the homeostasis of choroidal vascular function by promoting Bcl-2 like protein 11 (BIM), and its dysfunction led to exacerbation of CNV lesion. Therefore, this study suggests that the lncPNKY/PTBP1–miR-124 axis is involved in regulating the development of CNV, providing a potential therapeutic target for the treatment of CNV.

## Introduction

Choroidal neovascularization (CNV) is a process that occurs when choroidal endothelial cells grow and migrate from the choroid to the neuroretina and produce new blood vessels (Jager et al., 2008). This causes edema, bleeding, and damage to the photoreceptors due to exudation of intraretinal or subretinal fluid, hemorrhage, or fibrosis in the macula (Nowak, 2006). CNV is the characteristic pathological process of wet age-related macular degeneration (AMD), the leading cause of blindness in geriatric population. Moreover, in patients younger than 50 years of age, CNV can also be diagnosed in other ocular conditions such as trauma, angioid streaks, and high myopia (Weber and Heier, 2016). Its pathogenesis, likely multifactorial, involving a complex interaction of metabolic, functional, and genetic and environmental factors, remains poorly understood (Thomas et al., 2021). Current established medical treatments of CNV mainly include photodynamic therapy (PDT), thermal laser photocoagulation (TLP), transpupillary thermotherapy (TTT), and anti-vascular endothelial growth factor (VEGF) agents. Because of various evident pathogenic factors underlying the disease, available therapies can only help prevent further vision loss, but cannot cure or reverse the course of the disease to improve vision (Schmidt-Erfurth et al., 2014; Yang et al., 2016). Thus, further mechanisms and new treatments of CNV are urgently needed to be explored.

Long non-coding RNA (lncRNA) is a series of non-coding RNAs with a length longer than 200 nucleotides that do not encode functional proteins (Zhao et al., 2020). As the widest subgroup of ncRNAs, lncRNAs comprise various groups of transcripts and can be categorized into sense, antisense, bidirectional, intronic, and intergenic lncRNAs (Salehi et al., 2017). Depending on their subcellular localization, lncRNAs regulate gene expression at the epigenetic, transcriptional, and post-transcriptional levels by interacting with miRNA, mRNA, DNA, and protein (Agliano et al., 2019; Wu et al., 2020). The functional mechanisms of lncRNAs that have been confirmed include guiding the chromatin-modifying enzymes, epigenetic regulation of certain genes, decoying miRNAs through lncRNAs (miR sponges), and acting as molecular scaffolds (Peng et al., 2017). It was demonstrated that lncRNAs are important regulators of cellular differentiation, organogenesis, and tissue homeostasis. Moreover, the abnormal expression of some lncRNAs is involved in multiple pathological process and human diseases, such as cancer, diabetes, and ocular neovascularization (Batista and Chang, 2013; Cisse et al., 2019; Guo et al., 2019; Chen et al., 2020; Connerty et al., 2020; Kazimierczyk et al., 2020). Given the pivotal role of lncRNAs in gene regulation and tissue homeostasis, we speculate that lncRNAs play important roles in CNV.

LncRNA PNKY is an 825 nt, evolutionarily conserved, and nuclear-enriched lncRNA (Ramos et al., 2015). In this study, we investigated the role and the mechanism of PNKY in the pathological process of CNV. We showed that overexpression of PNKY ameliorated laser-induced choroidal neovascularization and cell damage induced by CoCl_2_. The interaction between PNKY and PTBP1 through which it modulates the function of downstream miRNAs and target proteins was identified. Thus, this study suggests that the intervention of PNKY could be a novel therapeutic strategy for neovascularization-related diseases.

## Materials and Methods

### Mice

The surgical specimens were handled according to the Declaration of Helsinki. Animal experiments were approved by the Animal Experiment Administration Committee of the Nanjing Medical University, and mice were handled according to the ARVO Statement. All mice were purchased from the Emeiling Animal Experimental Center of Nanjing Medical University. The mice were maintained under specific pathogen-free conditions on a 12-h light/dark cycle with free access to food and water at constant temperature (25°C) and relative humidity (60%).

### Intravitreal Injection

Mice were anesthetized with intraperitoneal ketamine (80 mg/kg) and xylazine (4 mg/kg), and pupils were dilated by phenylephrine (5 mg/ml) and tropicamide eye drops (5 mg/ml; Santen Pharmaceutical, Japan). After topically anesthetized with 0.5% proparacaine hydrochloride eye drops (Santen Pharmaceutical, Japan), 1.5 µl (1 × 10^12^ vg/ml) of adeno-associated virus (AAV) containing lncPNKY-shRNA or scrambled-shRNA was intravitreally injected using a 33-gauge needle under an operating microscope. AAV vectors were injected 2 weeks before laser injury due to the conversion of recombinant AAV-DNA to a transcriptionally active double-stranded form.

### Laser-Induced Choroidal Neovascularization (CNV)

The laser was used to induce choroidal neovascularization, as described previously (Lambert et al., 2013). In brief, mice were generally anesthetized, and the pupils were dilated with a drop of compound topiramate, followed by isobucaine hydrochloride as a topical anesthetic. Bruch’s membrane was then ruptured by laser photocoagulation using a 532-nm wavelength laser photocoagulator (75-µm spot size, 100-msec duration, and 100 mW) at 3, 6, 9, and 12 o’clock positions of the posterior pole around the optic disk, and a 10 mm coverslip was used to serve as a contact lens to view the retinal vessels. The formation of a small bubble which possesses a clear outline and no form of blurring or bleeding at the laser spot indicated a successful one. Seven days later, the eyes were harvested for immunohistochemical staining.

### IB4 Staining

After the fresh enucleated eyes were fixed in 4% paraformaldehyde (PFA) for 1 h at room temperature, the anterior segments were cut off and the choroid complexes (RPE/choroid/sclera) were flat-mounted to be permeabilized in PBS containing 0.5% Triton X-100 and Isolectin B4 (BSI-B4, L2895, Sigma, United States; 1:200) and were incubated at 4°C overnight. Images were taken using a fluorescence microscope and loaded into the ImageJ software to measure the CNV area. In brief, a thorough evaluation of the outline of each laser burn was carried out for the pixels within the evaluated area to be measured.

### HE Staining

For histopathological examination, anesthetized mice were subjected to heart perfusion with 40 ml 4% paraformaldehyde (PFA). Eyes were then fixed in Fekete’s fixative solution for 1 h and 4% PFA for 24 h, and later were embedded in paraffin wax. The samples were cut into coronal sections of 5-mm thickness and stained with Harris’ hematoxylin and eosin stain. The slides were stored with a few drops of PerMount mounting medium, dried overnight in the hood, and observed under a microscope.

### Explant Culture of Choroid

Sprouting of choroidal layers was tested as previously described (Shao et al., 2013). In brief, after the mice were anesthetized and then euthanized by cervical dislocation, their eyes were enucleated immediately and placed in an ice-cold complete medium, which was followed by the removal of corneas and lens. Then the RPE–choroid–sclera complexes were cut into pieces of about 1 mm × 1 mm fragments and inoculated in Matrigel^TM^ (BD Biosciences, Cat. 354230, 40 μl/well) coated in 24-well plates to be incubated without a medium for 10 min in a 37°C, 5% CO_2_ cell incubator in order for the Matrigel^TM^ to solidify. In addition, 500 ml of 10% FBS medium was added into each well and changed every 48 h.

### Cell Culture

RF/6A (Choroid-retinal endothelial cells; CRL-1780™, ATCC, United States) cells were cultured in Dulbecco’s Modified Eagle Medium (DMEM, Gibco BRL, United States) supplemented with 10% fetal bovine serum (FBS, ScienCell, United States) and antibiotics (100 U/ml penicillin and 100 μg/ml streptomycin; Gibco, United States). All cells were maintained at 37°C in an incubator containing 5% CO_2_ and 95% air.

### Cell Transfection

RF/6A cells were seeded in 24-well plates or 35 mm diameter dishes for 18–24 h before interference. According to manufacturer’s protocol, the cells were transfected with PNKY or NC siRNA at a concentration of 10 μM using a Lipo6000TM Transfection Reagent. Furthermore, 4–6 h after transfection, RF/6A cells were cultured in a fresh medium for 24–48 h before commencing other treatments.

### Plasmid Construction and Transfection

For the construction of the lncPNKY overexpression vector, PNKY cDNA was synthesized and cloned into a pcDNA3.0 vector. Transfection was carried out using Lipofectamine 3000 (Invitrogen), according to the manufacturer’s instructions.

### Cell Viability Assay

Cell viability was assessed by MTT [3-(4,5-dimethylthiazol-2-yl)-2, 5-diphenyltetrazolium bromide] assay. In brief, RF/6A cells were seeded into 96-well plates, and then transfected before incubating them with 100 µl of the sterile MTT labeling reagent (0.5 mg/ml; Sigma, M2128) at 37°C for 3 h. DMSO (150 μl/well, Sigma, W387520) was added into each hole and shook for 15 min in the dark. The absorbance (OD) at 450 nm was recorded and analyzed by a microplate reader (Molecular Devices).

### Cell Migration Assay

Cell migration and invasion were determined by the transwell assay. Polycarbonate transwell filters with 8.0-μm pores were inserted over the lower chambers. A total of 5 × 10^4^ cells suspended in 2% FBS were plated on the insert chamber supplemented with 600 μl of 10% FBS medium. After 12 h, cells were fixed and permeabilized in propyl alcohol for 15 min and then stained with 5% crystal violet stain overnight at 4°C. The number and morphology of cells were observed under an inverted microscope.

### Tube Formation Assay

Aliquots of 60 μl/well of the basement membrane matrix (BMM, BD Biosciences, San Jose, CA, United States) were coated into a cold pretreated 24-well plate to be incubated at 37°C, 5% CO_2_ for 1 h to coagulate. The pretreated RF/6A cells were resuspended and seeded on the BMM at a density of 2 × 10^4^ cells/well. After 6 h, the formation of tube-like structures was observed and captured with an Olympus (DB80; Tokyo, Japan) microscope.

### 5-Ethynyl-2′-deoxyuridine Immunofluoresence Assay

Cell proliferation was detected by EdU staining. RF/6A Cells were plated into 24-well plates at a density of 5 × 10^5^/hole and then transfected. The cells were incubated with 5-ethynyl-2′-deoxyuridine (EdU, C103310-3, RiboBio, China, 1:1,000) for 3 h before staining. Cell proliferation was detected using Apollo488 reaction cocktail (RiboBio, China), following the manufacturer’s instructions. Cell nuclei were marked with Hoechst 33342 stain (RiboBio, China, 1:1,000) for 30 min. Percentage of proliferative cells was determined by blinded quantitation of EdU-positive cells under an Olympus (DB80; Tokyo, Japan) microscope.

### Calcein-AM and Propidium Iodide Double Staining

RF/6A cells were stimulated to apoptosis with 200 μM cobalt chloride (CoCl_2_) for 24 h. Then cells were stained with Calcein-AM solution (10 μmol/l; Molecular Probes, United States) and PI (10 μmol/l; Molecular Probes, United States). Excitation filter of 490, 545, and 347 nm were used to detect the living cells and the dead cells for fluorescence observation.

### Dual Luciferase Activity Assay

A dual luciferase activity assay was conducted according to the manufacturer’s instructions. In brief, pGL3 vectors (Promega, Madison, WI, United States) were constructed in advance with 3′-UTR or mutant 3′-UTR of BIM or PNKY containing the putative target site for miR-124 inserted into the downstream of the luciferase gene. Then RF/6A cells were transfected by prefabricated pGL3-vectors along with miR-124 mimic after seeding in 24-well plates at the concentration of 2 × 10^5^ cells/well. After 24 h, a Dual Luciferase Reporter Assay System (Promega) was used to detect the relative luciferase activity.

### Quantitative Real-Time PCR

Total RNA was extracted using the TRIzol reagent (15596026, Invitrogen, United States) from RF/6A cells, according to the protocol. Then aliquots of RNA were reversely transcribed by specific primers and SYBR green Master Mix (A25741, Thermo Fisher Scientific, United States). A quantitative real-time PCR was carried out using specific primers in the PikoReal Real-Time PCR System (Thermo Fisher Scientific). The reaction was performed as follows: initial denaturation at 94°C for 5 min; 30 cycles of amplification (94°C for 30 s, 54°C for 30 s, and 72°C for 1 min); and final extension at 72°C for 3 min. Relative gene expression was determined by the 2-ΔΔCt methods. All qRT-PCR reactions were performed in triplicate and averaged.

### Clinic Inclusion and Exclusion Criteria

For wet AMD patients: 1) we clearly visualized growing neovascularization within the macula by optical coherence tomography angiography (OCTA). OCTA can clearly display distinct layers of retinal vessels from inside to outside. CNV lesions were mainly in the outer retina and choriocapillaris layers (Supplementary Figure S2A). The thickness and growth layers of CNV could be seen in transverse and longitudinal plane of the lesion site (Supplementary Figure S2B). In addition, we observed the vascular density and tissue thickness of different parts of the retina by OCTA (Supplementary Figure S2C). 2) They never received anti-VEGF intraocular injection therapy. 3) Patients with systematic diseases, malignant tumors of any location, and other ocular diseases were excluded from the study.

For age-related cataract (ARC) and glaucoma patients: 1) patients who are clinically diagnosed with ARC or glaucoma and will undergo surgical treatment were selected. 2) Patients with systematic diseases, malignant tumors of any location, and other ocular diseases, especially AMD, were excluded from the study.

### Statistical Analysis

All results are presented as mean ± SEM unless otherwise stated. The normal distribution of data was evaluated by the normality tests. For normally distributed data with equal variance, the difference was analyzed by two-tailed Student’s *t* test (two group comparisons) and analysis of variance (ANOVA), followed by post hoc Bonferroni’s test (multigroup comparisons) as appropriate. For non-normally distributed data or data with unequal variances, the difference was analyzed by the non-parametric Mann–Whitney *U* test (two group comparisons) or Kruskal–Wallis test, followed by post hoc Bonferroni’s test (multigroup comparisons). The results were considered significantly different if *p* value was <0.05.

## Results

### LncRNA PNKY Expression is Downregulated in Laser-Induced CNV and Endothelial Cells Upon Hypoxic Stress

**FIGURE 1:**
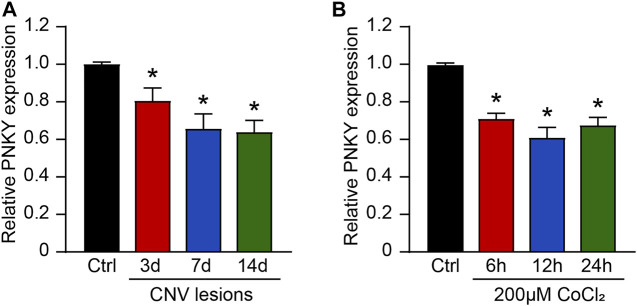
LncRNA PNKY expression pattern in CNV lesions and in RF/6A cells under the hypoxic condition. **(A)** qRT-PCRs were executed to detect the level of PNKY in the choroidal samples of C57BL/6 mice after 3, 7, and 14-day laser photocoagulation (*n* = 4, *p < 0.05, Kruskal–Wallis’ test followed by Bonferroni’s post hoc test). **(B)** RF/6A cells were incubated with 200 μM CoCl_2_ for the specified time. qRT-PCRs were used to detect PNKY expression (*n* = 4, *p < 0.05, one-way ANOVA followed by Bonferroni’s post hoc test).

We first determined whether lncRNA PNKY expression is altered during choroidal vascular dysfunction condition. In the mouse model of laser-induced CNV, the results of qRT-PCR assays showed that PNKY was significantly downregulated in the RPE/choroid complexes on day 3, day 7, and day 14 after laser photocoagulation (Figure 1A). Hypoxia is one of the most well-known angiogenic factors in CNV, and choroidal endothelial cells play key roles in the development of CNV. We then investigated whether PNKY expression is altered in endothelial cells in the hypoxic condition. Choroid-retinal endothelial cells (RF/6A) were exposed to CoCl_2_ for 6, 12, and 24 h to mimic hypoxic stress. PNKY expression was significantly downregulated in RF/6A cells in a time-dependent manner (Figure 1B). Collectively, these results provide the evidence that PNKY is a potential regulator of diabetic choroidal neovascularization.

### LncRNA PNKY Regulates CNV Development *In Vivo*

**FIGURE 2:**
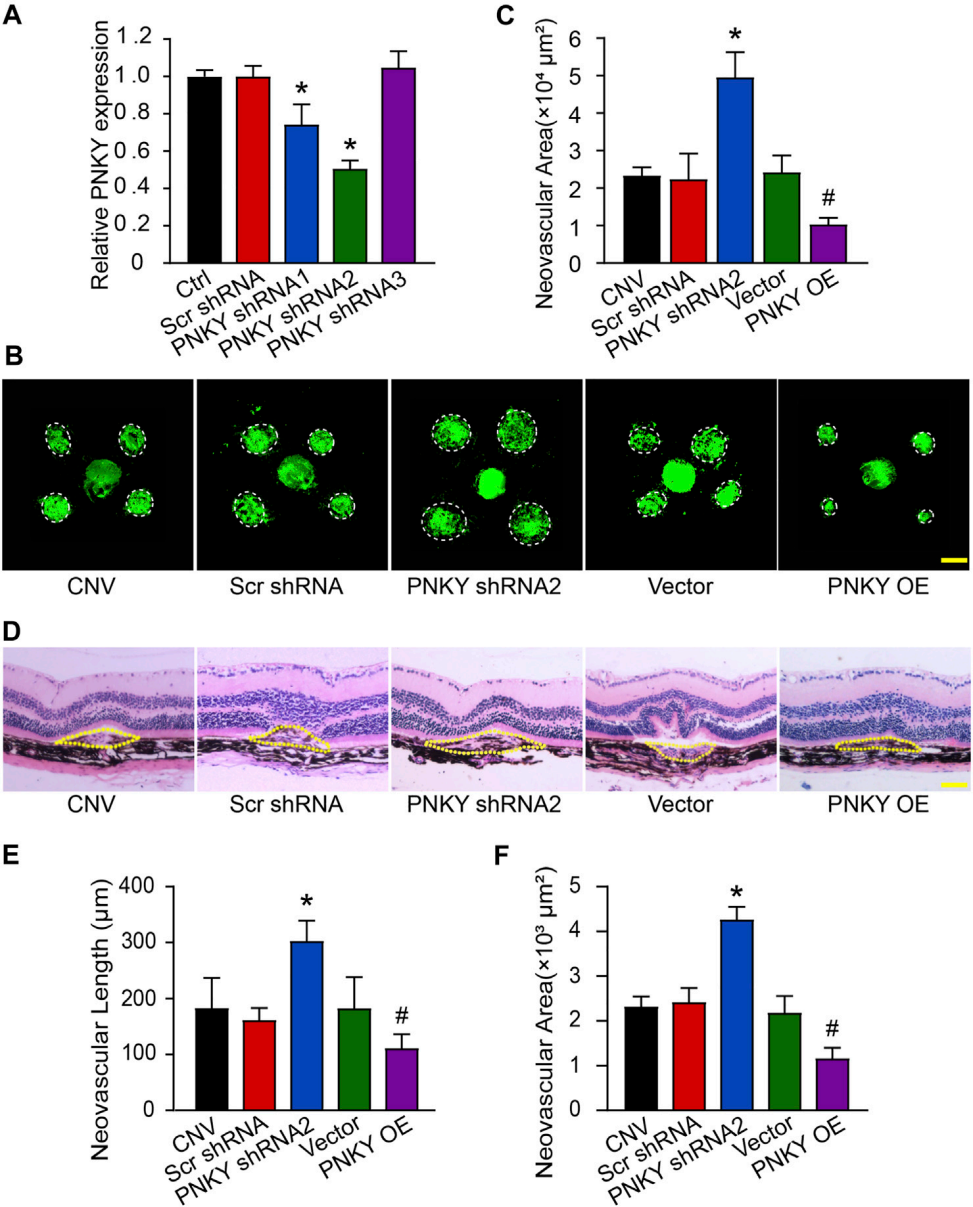
LncRNA PNKY silencing promotes the development of laser-induced CNV *in vivo*. **(A)** Eight-week-old C57BL/6 mice were injected with scrambled (Scr) shRNA, PNKY shRNA, or left untreated (Ctrl). qRT-PCRs were implemented to detect the expression of PNKY in the choroid at day 14 (*n* = 5). **(B,C)** Eight-week-old C57BL/6 mice received an intravitreal injection of scrambled (Scr) shRNA, PNKY shRNA2, vehicle (Vector), PNKY overexpression vector (PNKY OE), or left untreated (CNV) 14 days before laser photocoagulation. CNV in flattened choroidal organizations received visualization by fluorescent labeling of IB4 and quantification on day 7. The lesion regions were highlighted by white circles (Scale bar, 100 μm, *n* = 5). **(D)** Seven days after photocoagulation, typical images of hematoxylin and eosin staining of cross sections of the CNV were displayed and quantification of the length **(E)** and the area **(F)** of the CNV was performed. Yellow dashed lines delineate the lesions (Scale bar, 100 μm, *n* = 5). All significant difference was evaluated by Kruskal–Wallis’s test followed by post hoc Bonferroni’s test. *p < 0.05 versus Scr shRNA, #*p* < 0.05 versus Vector.

To investigate the role of PNKY in the development of CNV *in vivo*, three optional adeno-associated viral shRNAs were designed for PNKY silencing. PNKY shRNA injection significantly decreased choroidal PNKY. We selected shRNA2 for PNKY silencing because it had the best gene silencing efficiency. We also constructed the PNKY overexpression vector by synthesizing and cloning PNKY into the pcDNA3.0 vector (Figure 2A).

Isolectin B4 (IB4) immunofluorescence staining revealed that PNKY silencing led to an increased neovascular area in the choroidal flat mounts compared with the Scr shRNA-injected group. In contrast, PNKY overexpression decreased the neovascular area compared with mice injected with the vector (Figures 2B,C). Next, we conducted a HE staining analysis to examine the CNV lesions in area and length. The results showed that PNKY silencing promoted increases in CNV lesions in both area and length, while PNKY overexpression protected the CNV lesions by reducing neovascularization (Figures 2D–F). These findings suggest that PNKY regulates CNV development *in vivo*.

### LncRNA PNKY Regulates Choroidal Sprouting *Ex Vivo*

**FIGURE 3:**
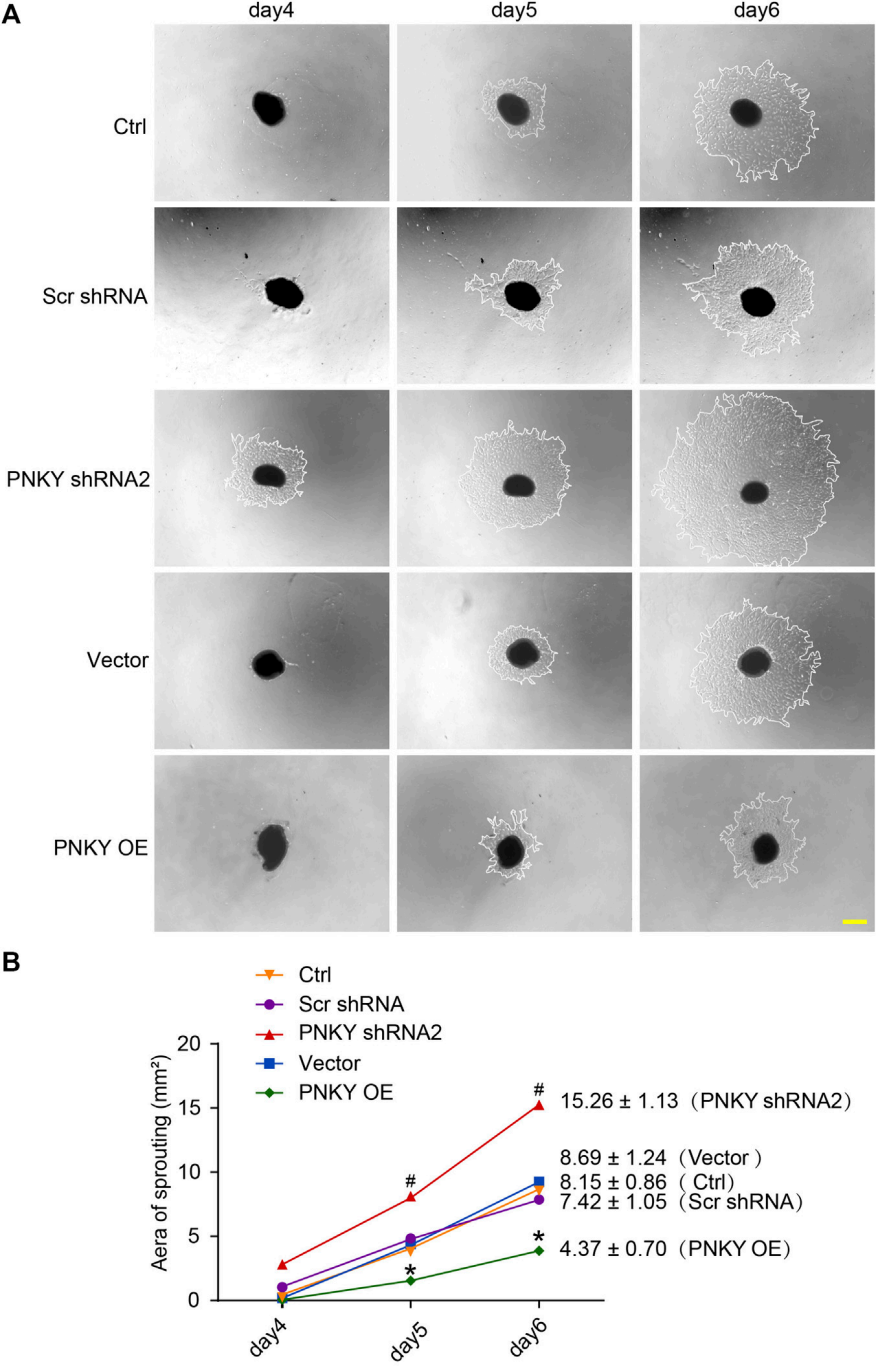
LncRNA PNKY regulates choroidal sprouting *ex vivo*. Eight-week-old C57BL/6 mice received an intravitreal injection of scrambled (Scr) shRNA, PNKY shRNA2, vehicle (Vector), PNKY overexpression vector (PNKY OE), or left untreated (Ctrl). At day 14, the mouse RPE/choroid complexes were dissected. The peripheral regions were cut into 1 mm × 1 mm pieces and seeded into the 24-well plates to assess the potential angiogenic capacity of choroidal explants in each group of mice. **(A)** Example images of the choroidal sprouting areas at specified time points were demonstrated. Scale bar, 500 μm. **(B)** Quantification of the areas of the CNV sprouts was shown (*n* = 5, *p < 0.05 versus Scr shRNA, #*p* < 0.05 versus Vector, Kruskal–Wallis’ test followed by Bonferroni’s post hoc test).

We then determined the effect of PNKY on choroidal angiogenic activity using the choroidal sprouting assay, an *ex vivo* model of microvascular proliferation. The experiments were divided into five groups: PNKY silencing group, PNKY overexpression group, scrambled shRNA group, vector group, and untreated group. The choroidal explants were embedded in Matrigel, and the sprouting areas of these explants were photographed on day 4, 5, and 6. The results showed that PNKY silencing led to increased sprouting ability of choroidal ECs, while PNKY overexpression led to decreased sprouting ability (Figure 3A). Figure 3B showed the quantitative results of choroidal sprouting areas. These results indicate that PNKY regulates the development of CNV *ex vivo*.

### LncRNA PNKY Regulates Endothelial Cell Function *In Vitro*

**FIGURE 4:**
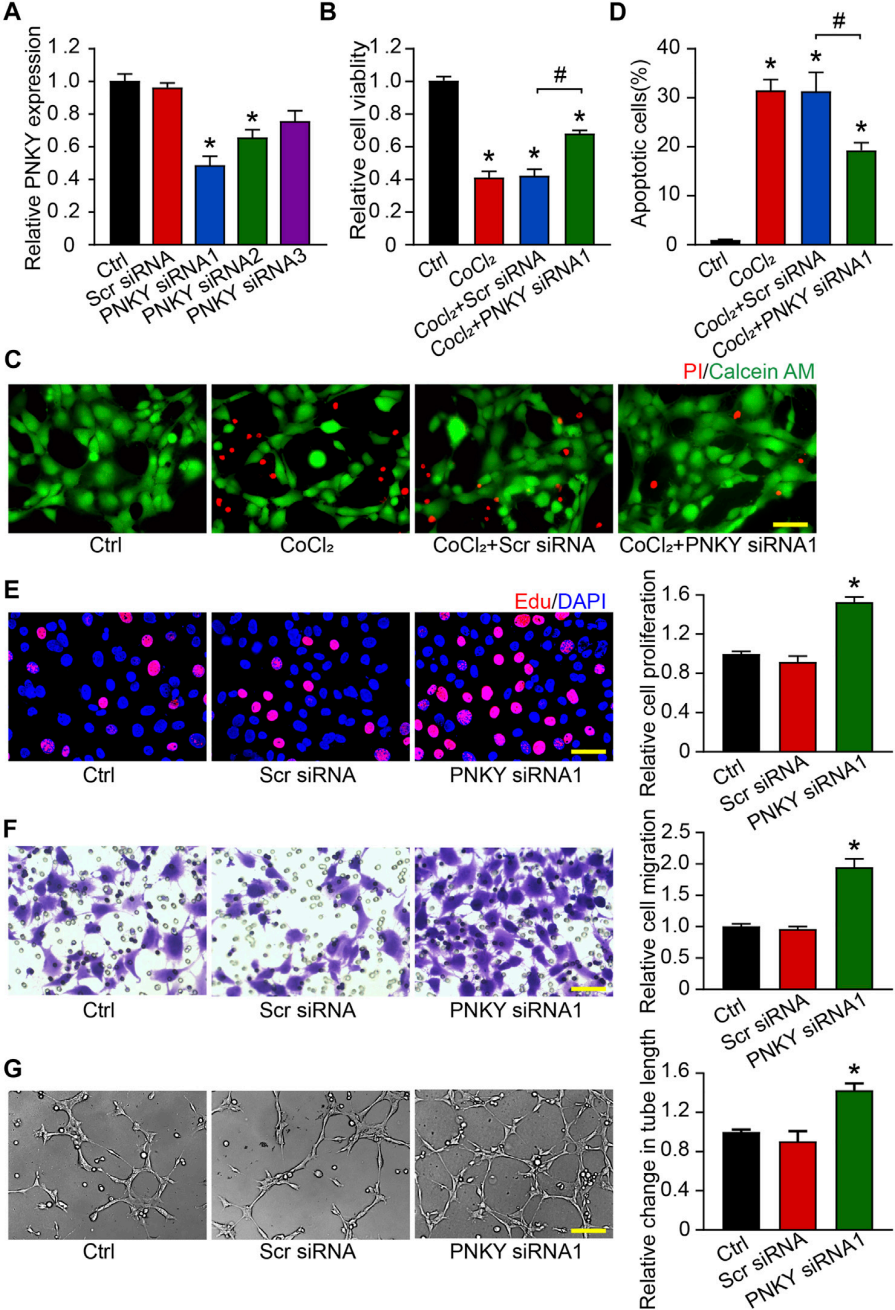
LncRNA PNKY regulates endothelial function *in vitro*. RF/6A cells were transfected with scrambled (Scr) siRNA, PNKY siRNA1, or left untreated (Ctrl) for 24 h before further processed. **(A)** qRT-PCRs were conducted to detect PNKY expression (*n* = 4). RF/6A cells were exposed to 200 μM CoCl_2_ to mimic hypoxic stress for 24 h. **(B)** Cell viability was detected using MTT method (*n* = 4, *p < 0.05 versus Ctrl). **(C,D)** The group without CoCl_2_ treatment was taken as the control group. Apoptotic cells were detected by PI/Calcein-AM staining. Green: live cells; red: dead or dying cells (Scale bar, 50 μm, *n* = 4, *p < 0.05 versus Ctrl). **(E)** EdU detection kit (RiboBio, Guangzhou, China) was used to test cell proliferation by analyzing the incorporation of EdU in DNA synthesis. Blue: DAPI; red: EdU (Scale bar, 50 μm, *n* = 4). **(F)** Transwell assay was performed to measure migration of RF/6A cells. Quantification revealed the cells migrating through the transwell (Scale bar, 50 μm, *n* = 4). **(G)** To observe the tube-like structures, RF/6A cells were seeded on the Matrigel matrix. Furthermore, 4 h after cell seeding, average length of tube formation for each field was statistically analyzed (Scale bar, 200 μm, *n* = 4). All significant difference was determined by one-way ANOVA followed by Bonferroni’s post hoc test. *p < 0.05 versus Scr siRNA, #*p* < 0.05 between the marked groups.

To investigate the role of PNKY in the development of CNV *in vitro*, RF/6A cells were transfected with PNKY small interfering RNA (siRNA). Three PNKY small interfering RNAs (siRNA) were designed for PNKY silencing. siRNA1 showed the greatest silencing efficiency and was selected for subsequent experiments (Figure 4A).

In response to hypoxia stress, PNKY knockdown significantly increased RF/6A cells viability (Figure 4B) and protected RF/6A cells against apoptosis as shown by decreasing PI-positive cells (Figures 4C,D). Under basal conditions, EdU staining revealed that PNKY silencing increased RF/6A cells’ proliferation (Figure 4E), and transwell and Matrigel tube formation assays revealed that PNKY silencing by siRNA1 significantly accelerated the migration (Figure 4F) and tube formation (Figure 4G) of RF/6A cells. Conversely, PNKY overexpression attenuated the viability and proliferation and decelerated the migration and tube formation of RF/6A cells (Supplementary Figure S1). Taken together, these results indicate that PNKY regulates RF/6A cell functions *in vitro*.

### LncRNA PNKY Functions Biologically by Binding to PTBP1

An increasing probe indicated that lncRNAs may fulfill their functions of regulating gene expression through binding to their target proteins (Wang and Chang, 2011). Lnc tarD and LncSEA analysis databases indicated that the RNA-binding protein polypyrimidine bundle-binding protein 1 (PTBP1) can specifically bind to PNKY to exert biological interactions. PTBP1 is a widely expressed RNA-binding protein that is closely associated with RNA metabolism as a regulator of post-transcriptional gene expression (Sawicka et al., 2008). In addition to RNA splicing, PTBP1 can also antagonize the action of miRNA by binding in the 3′UTRs of transcripts (Takahashi et al., 2015).

**FIGURE 5:**
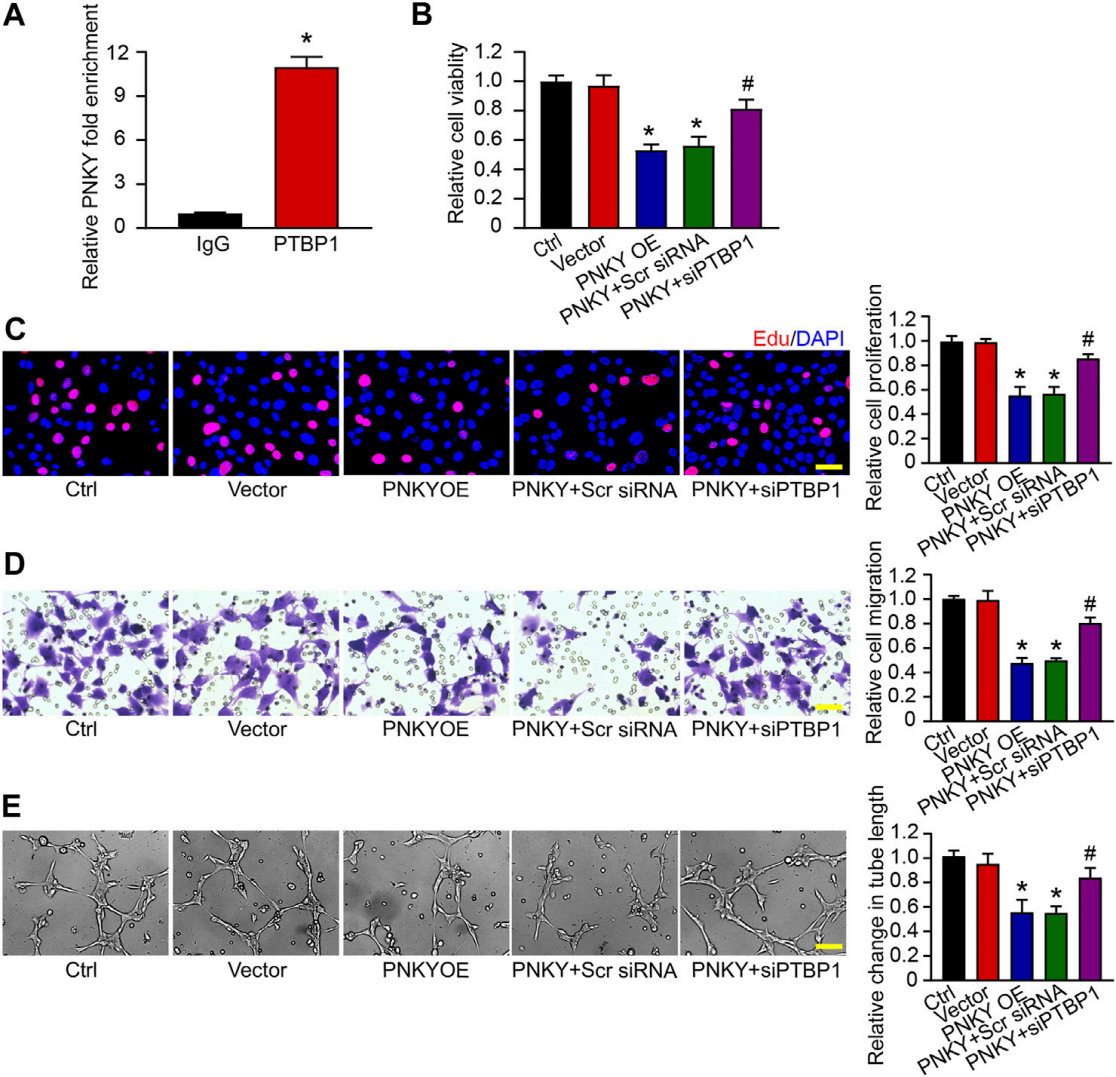
LncRNA PNKY functions biologically by binding to PTBP1. **(A)** PTBP1 antibody was incubated with RF/6A cell extracts and qRT-PCR was performed to test the relative fold enrichment of PNKY associated with PTBP1. Normalized to the negative IgG control (*n* = 4, *p < 0.05 versus IgG, Mann–Whitney *U* test). RF/6A cells were transfected with pcDNA3.0 vector (Vector), pcDNA3.0-PNKY, pcDNA3.0-PNKY plus scrambled (Scr) siRNA, pcDNA3.0-PNKY plus PTBP1 siRNA (siPTBP1), or left untreated (Ctrl) for 24 h. **(B)** Cell viability was detected using MTT method (*n* = 4, *p < 0.05 versus Vector, #*p* < 0.05 versus PNKY OE, one-way ANOVA followed by Bonferroni’s post hoc test). **(C)** Cell proliferation was detected using EdU staining and quantified. Blue: DAPI; red: EdU (Scale bar, 50 μm, *n* = 4, *p < 0.05 versus Vector, #*p* < 0.05 versus PNKY OE, one-way ANOVA followed by Bonferroni’s post hoc test). **(D)** The migration of RF/6A cells was detected using Transwell assay and quantified (Scale bar, 50 μm, *n* = 4, *p < 0.05 versus Vector, #*p* < 0.05 versus PNKY OE, one-way ANOVA followed by Bonferroni’s post hoc test). **(E)** The pictures of tube-shaped structures were captured 4 h after cells seeding on the matrix to analyze the average length of tube formation for each field (Scale bar, 200 μm, *n* = 4, *p < 0.05 versus Vector, #*p* < 0.05 versus PNKY OE, one-way ANOVA followed by Bonferroni’s post hoc test).

We first conducted RNA immunoprecipitation (RIP) using anti-PTBP1 antibodies, followed by qRT-PCR, to test whether endogenous PNKY could bind PTBP1 protein in RF/6A cells. The results showed that PNKY was significantly enriched in samples immunoprecipitated with anti-PTBP1, as compared with negative IgG control (Figure 5A).

Then we probed the effect of lncRNA PNKY–PTBP1 interaction on RF/6A cells *in vitro*. The MTT assay showed that PNKY overexpression inhibited cell viability and PTBP1 silencing blocked the depression (Figure 5B). EdU staining, transwell, and Matrigel tube formation assays demonstrated that PNKY upregulation suppressed cell proliferation (Figure 5C), migration (Figure 5D), and tube formation activity (Figure 5E), whereas when PTBP1 was downregulated, the inhibitory effect of PNKY cannot be exerted. Collectively, these results suggest that lncRNA PNKY regulates the function of RF/6A cells through binding to PTBP1.

### PNKY–PTBP1 Interaction Functions by Blocking miRNA-124 Biogenesis in CNV Development

**FIGURE 6:**
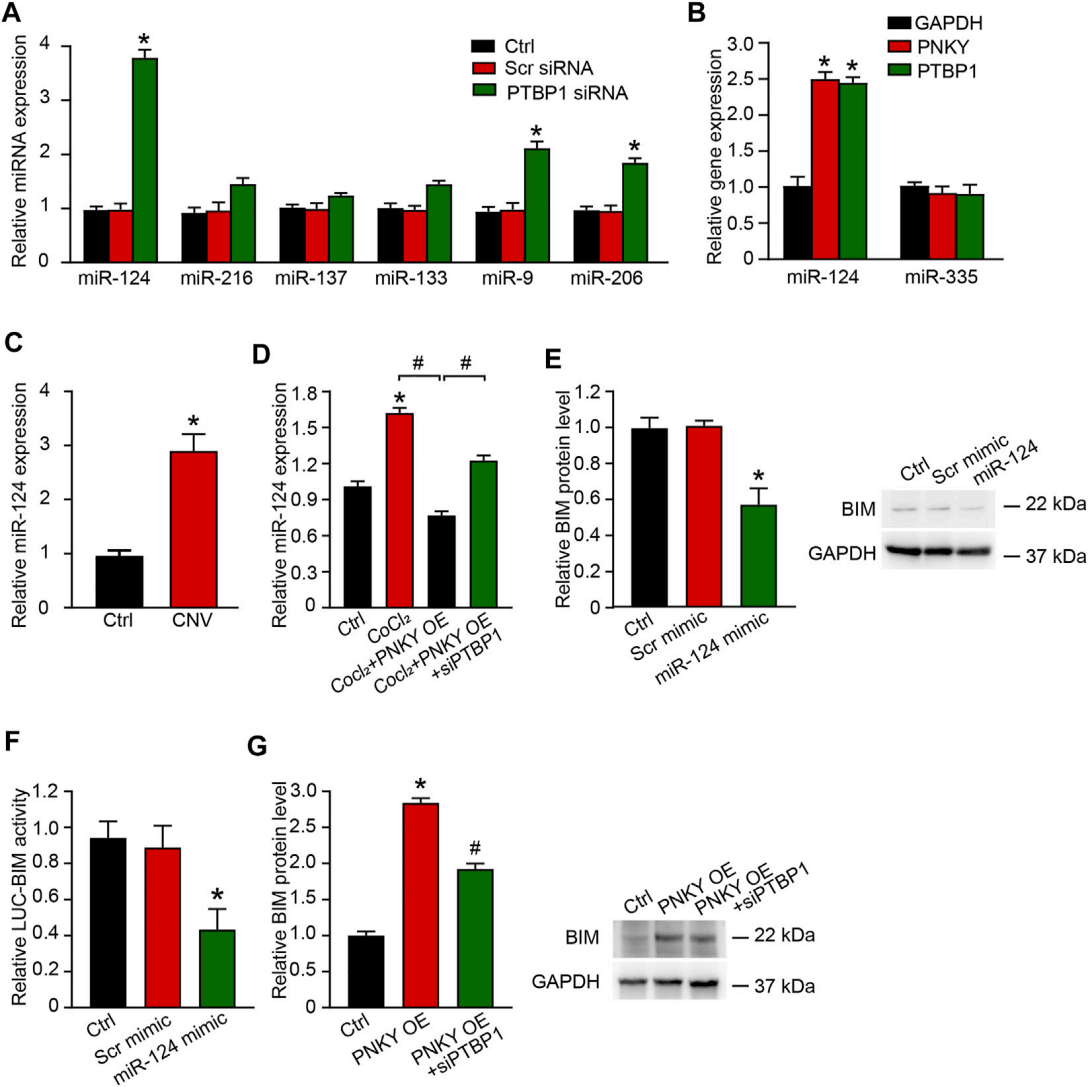
PNKY–PTBP1 interaction functions by blocking miRNA-124 biogenesis in CNV development. **(A)** RF/6A cells were transfected with scrambled (Scr) siRNA, PTBP1 siRNA, or left untreated (Ctrl) for 24 h. qRT-PCRs were performed to detect miRNAs expression (*n* = 4, *p < 0.05 versus Scr siRNA, one-way ANOVA followed by Bonferroni’s post hoc test). **(B)** RF/6A cells were transfected with the 3′-end biotinylated miRNA duplexes for streptavidin capture. qRT-PCRs were performed to detect the levels of PNKY, PTBP1, and GAPDH in the input and bound fractions and the relative immunoprecipitate (IP)/input ratios were plotted. **(C)** qRT-PCRs were conducted to detect the expression of miR-124 in CNV lensions of C57BL/6J mice (*n* = 6 animals per group, *p < 0.05, Mann–Whitney *U* test). **(D)** RF/6A cells were exposed to 200 μM CoCl_2_ or left untreated (Ctrl) for 24 h. RF/6A cells under hypoxic stress were transfected with pcDNA3.0-PNKY, pcDNA3.0-PNKY plus PTBP1 siRNA, or left untreated (CoCl_2_) for 24 h. qRT-PCRs were conducted to detect the expression of miR-124 (*n* = 4, *p < 0.05 versus Ctrl, #*p* < 0.05 versus PNKY OE, one-way ANOVA followed by Bonferroni’s post hoc test). **(E)** RF/6A cells were transfected with Scr mimic, miR-124 mimic, or left untreated (Ctrl). qRT-PCRs were conducted to detect BIM expression (*n* = 4, *p < 0.05 versus Scr mimic, one-way ANOVA followed by Bonferroni’s post hoc test). **(F)** RF/6A cells were co-transfected LUC-BIM with miRNA mimics. Luciferase activity was detected 36 h after transfection (*n* = 4, *p < 0.05 versus Scr mimic, one-way ANOVA followed by Bonferroni’s post hoc test). **(G)** RF/6A cells were transfected with pcDNA3.0-PNKY, pcDNA3.0-PNKY plus PTBP1 siRNA, or left untreated (Ctrl) for 24 h. qRT-PCRs were conducted to detect BIM expression (*n* = 4, *p < 0.05 versus Ctrl, #*p* < 0.05 versus PNKY OE, one-way ANOVA followed by Bonferroni’s post hoc test).

We continued to analyze the downstream molecular events of PNKY–PTBP1 regulating CNV development. We analyzed the publicly available Targetscan database and finally focused on PTBP1 target microRNAs, miR-124, miR-216, miR-137, miR-133, miR-9, and miR-206. qRT-PCR showed that miR-124, miR-9, and miR-206 were statistically upregulated in PTBP1 silenced groups compared to the controls (Figure 6A). Considering that miR-124 has been previously reported functional mutation associated to retinal neovascularization (Yin et al., 2018; Xia et al., 2021), we hypothesized that PNKY–PTBP1 interaction functions in CNV development by targeting miR-124.

Using the biotin-coupled miR-124, we showed high level enrichment of PNKY and PTBP1 in miR-124-captured fraction compared to the negative control, biotinylated miR-335 (Figure 6B). Using qRT-PCR, we showed that in laser-induced CNV lesions, the miR-124 expression level was significantly elevated (Figure 6C). In RF/6A cells under hypoxic stress, miR-124 expression was increased. Upregulating PNKY repressed this increase and PTBP1 silencing eliminated the effect of PNKY (Figure 6D). Considering that no clear miR-124 binding sites were found on the PNKY sequence, PNKY altered the messenger miRNA by interacting with RNA binding protein PTBP1 but not directly targeting miR-124. The interaction constituted the regulation network.

The Targetscan database was then used to predict the target genes of miR-124. The candidate gene BIM (Bcl-2-like 11) was identified, which has been reported to promote cell apoptosis under both physiological and pathological conditions and inhibit cell proliferation and differentiation (Youle and Strasser, 2008). miR-124 mimic transfection downregulated BIM expression in RF/6A cells (Figure 6E). miR-124 mimic transfection decreased the luciferase activities of reporter constructs containing target sequences of BIM (Figure 6F). PNKY promoted the expression of BIM, while PTBP1 silencing reversed the augmentation (Figure 6G).

### PNKY–PTBP1/miR-124 Interaction Regulates the Function of RF/6A Cells

**FIGURE 7:**
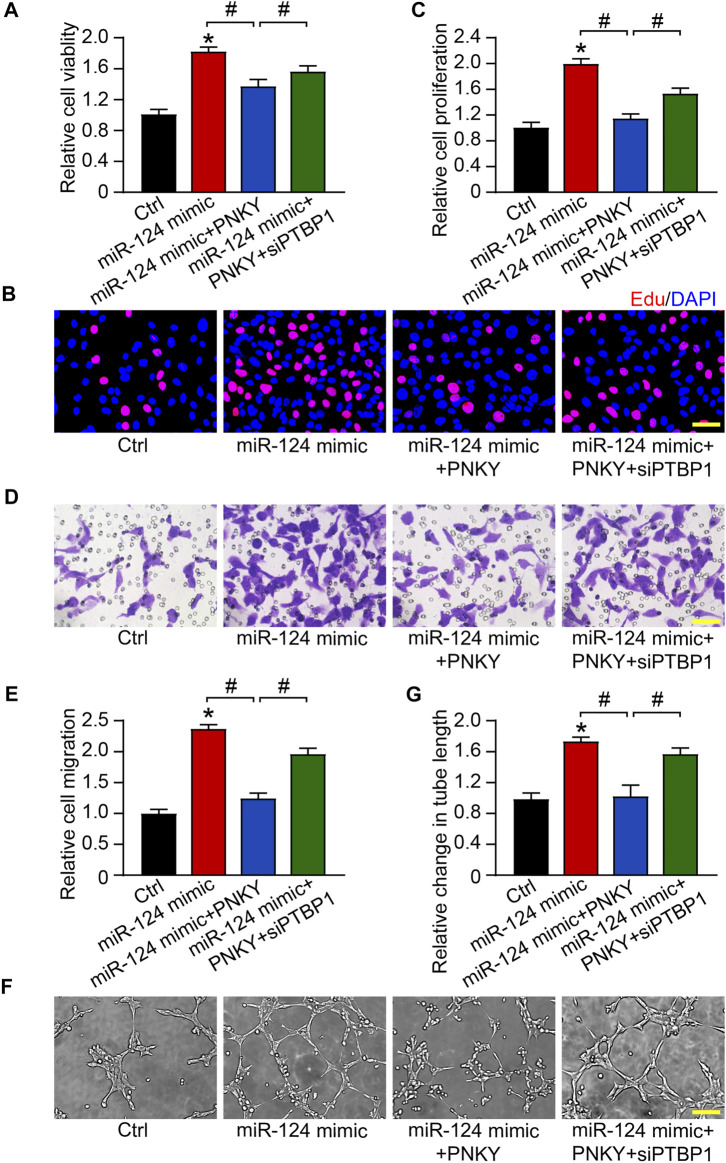
PNKY–PTBP1/miR-124 interaction regulates the function of RF/6A cells. RF/6A cells were transfected with scrambled miR-124 mimic, miR-124 mimic plus pcDNA3.0-PNKY, miR-124 mimic plus pcDNA3.0-PNKY and PTBP1 siRNA, or left untreated (Ctrl) for 24 h before further processed. **(A)** Cell viability was determined using MTT method (*n* = 4). **(B,C)** Cell proliferation was detected with EdU staining and quantified. Blue: DAPI; red: EdU (Scale bar, 50 μm, *n* = 4). **(D,E)** The migration of RF/6A cells was detected using Transwell assay and quantified (Scale bar, 50 μm, *n* = 4). **(F,G)** The tube-like structures were observed 4 h after cell seeding on the matrix, and the average length of tube formation for each field was statistically analyzed (Scale bar, 200 μm, *n* = 4). All significant difference was determined by one-way ANOVA followed by Bonferroni’s post hoc test. *p < 0.05 versus Ctrl, #*p* < 0.05 between the marked groups.

We verified the regulatory effects of miR-124 on RF/6A cells *in vitro*. The MTT assay showed that miR-124 upregulation promoted the viability of RF/6A cells. PNKY overexpression reversed the trend and PTBP1 silencing turned the response in the opposite direction (Figure 7A). EdU staining, transwell, and Matrigel tube formation assays revealed increased miR-124 enhanced cell proliferation (Figures 7B,C), migration (Figures 7D,E), and tube formation activity (Figures 7F,G). PNKY overexpression abolished the promotion and PTBP1 silencing further repealed the impact of PNKY. The results systemically indicate that PNKY-PTBP1/miR-124 interaction is involved in regulating endothelial cell function *in vitro*.

### Clinical Relevance of lncRNA PNKY in the Patients With CNV

**FIGURE 8:**
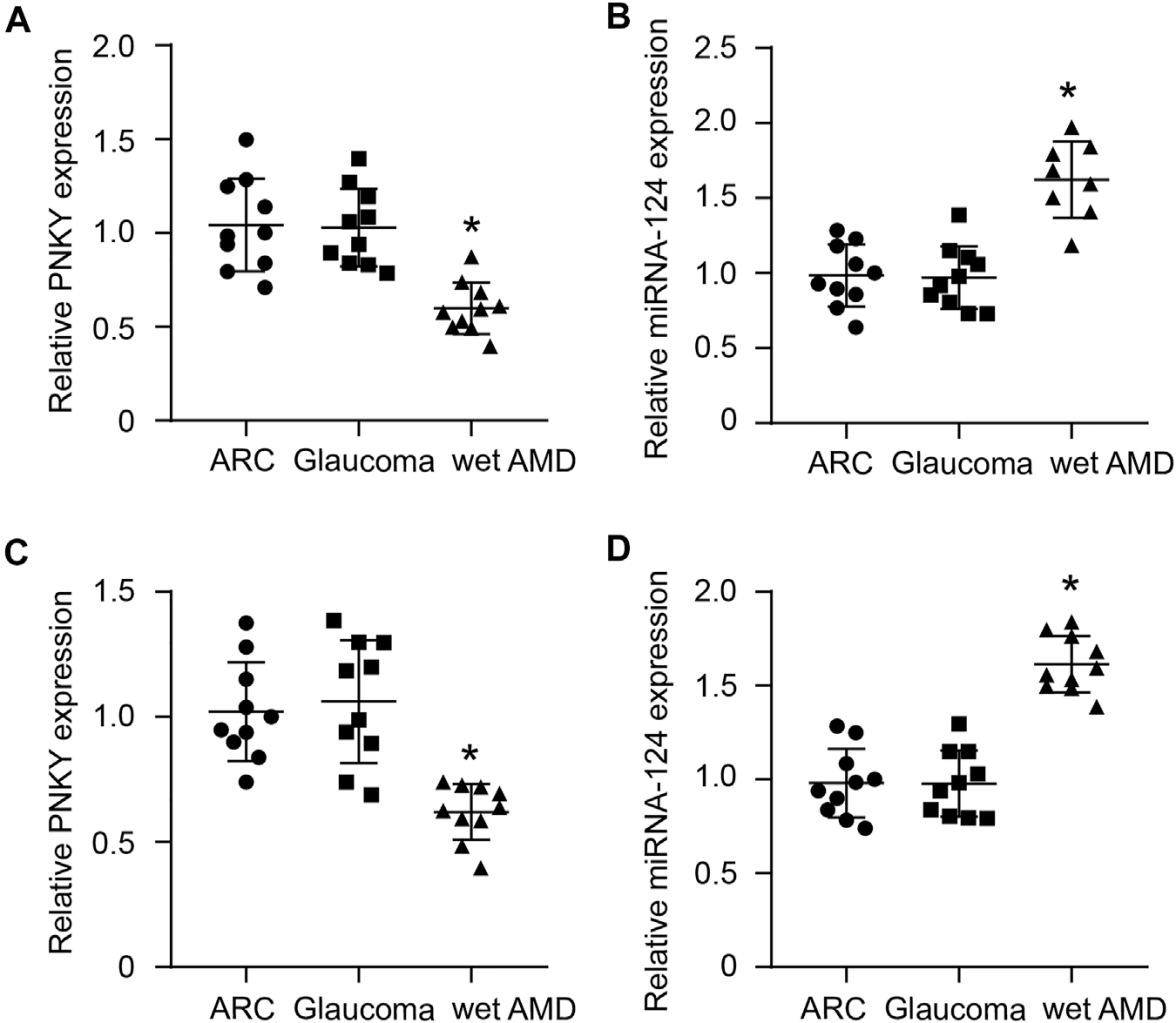
Clinical relevance of cZBTB44 in CNV patients. **(A,B)** qRT-PCRs were performed to detect the level of PNKY and miR-124 in the AH of patients with ARC, glaucoma, and wet AMD. **(C,D)** qRT-PCRs were conducted to detect the expression of PNKY and miR-124 in the plasma fraction of patients with ARC, glaucoma, and wet AMD (*n* = 10, *p < 0.05 versus ARC and Glaucoma, Kruskal–Wallis’ test followed by Bonferroni’s post hoc test).

We subsequently investigated the clinical implication of dysregulated PNKY/PTBP1–miR-124 signaling. The aqueous humor (AH) samples and plasma fraction from the patients with age-related cataract (ARC) or glaucoma or wet AMD were collected and sequenced by qRT-PCR. The results showed that PNKY expression was downregulated (Figures 8A,B), while miR-124 was upregulated in the AH and plasma fraction of patients with wet AMD but no decrease in that of patients with ARC and glaucoma (Figures 8C,D).

**FIGURE 9:**
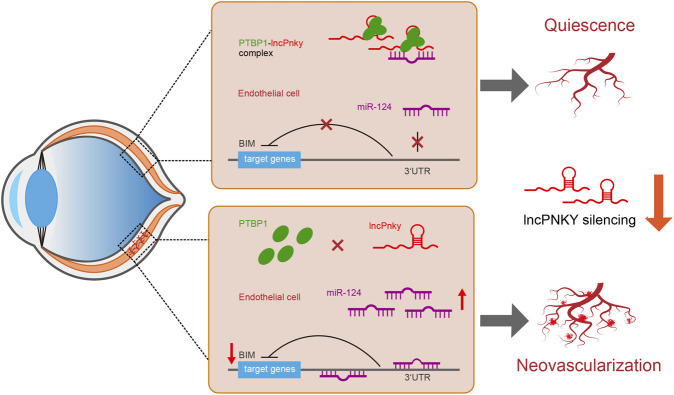
Schematic illustration of the mechanism hypothesis. We hypothesized that lncRNA PNKY is an inhibitory factor for cell differentiation and growth *in vivo*. When PNKY is silenced, the derepression of miR-124 restrains the expression of the target gene BIM, which results in decreased apoptosis, increased proliferation, and aggravated lesions.

Collectively, PNKY/PTBP1–miR-124 crosstalk regulates CNV development and PNKY is potentially involved in the pathogenesis of CNV. When PNKY is silenced, the derepression of miR-124 restrains the expression of the target gene BIM, which results in decreased apoptosis, increased proliferation, and aggravated lesions. Figure 9 summarizes the findings of this study and the putative molecular mechanisms involved.

## Discussion

According to the Human Genome Project, only 1–2% of human genome can encode proteins, leading non-coding RNA to be acknowledged as non-functional genetic noise for a time (Lander et al., 2001). Along with the advances of genomic sequencing technologies and functional assays, people gradually realized the function of non-coding RNA and sorted it by length (Palazzo and Lee, 2015; Hombach and Kretz, 2016). LncRNAs are series of non-coding RNAs with a length longer than 200 nucleotides that do not encode functional proteins (Zhao et al., 2020). These RNAs regulate the gene expression positively or negatively through epigenetic and post-transcriptional regulation by targeting miRNAs. Aberrant lncRNA expression has been associated with various diseases, such as tumor, hypertension and diabetes (Cisse et al., 2019; Guo et al., 2019; Chen et al., 2020; Connerty et al., 2020; Kazimierczyk et al., 2020). In our study, we illustrated that PNKY expression was downregulated in laser-induced CNV mouse model and in RF/6A cells upon hypoxia stress. PNKY silencing facilitated CNV development *in vivo*, promoted choroidal explant sprouting in the choroidal capillary angiogenesis model, and enhanced the angiogenic properties of RF/6A cells. We also explored the regulatory mechanism of PNKY in CNV development. PNKY, first, bound to RBP, then inhibited the activity of miR-124, and ultimately upregulated the expression of antiproliferative BIM. This study provides a potential pathogenesis and treatment target for CNV.

CNV is a typical pathological process of neovascularization associated with inflammation and oxidative stress which is always involved in ocular diseases including wet AMD, high myopia, and central serous chorioretinopathy (CSC). This pathological process is regulated in the same way as the regulation of angiogenesis occurring in other systemic diseases, such as strokes and tumors, providing an easily accessible window for studying neovascular diseases. Furthermore, *in vivo* genome editing therapy is an active topic in recent research works, the eye’s relative independence and immune privilege make it become an ideal organ for the genetic study (Hung et al., 2016; Huang et al., 2017; Yu et al., 2017). Thus, we conducted the mouse model of CNV induced by laser burning. PNKY expression was downregulated in CNV lesions and PNKY silencing was augmented, while overexpression suppressed the neovascularization.

Sprouting is the frontline of angiogenesis and contributes to the development of CNV. The choroidal sprouting is a quintessential assay for microangiogenesis study appeared in recent years, which is analogous to the aortic ring assay for large vessel angiogenesis (Shao et al., 2013; Kim et al., 2017). We introduced the sprouting model to study the regulation of PNKY in CNV development *ex vivo*. PNKY silencing promoted the choroidal explant sprouting. By contrast, PNKY overexpression inhibited the choroidal explant sprouting. These results indicate that PNKY regulates the choroidal explant sprouting *ex vivo*.

The vascular endothelium is a barrier inside and outside the blood vessel which limits the physiological exchange of substances between the blood and surrounding tissue fluids. Thus, it is vital for maintaining bodily homeostasis and preventing leakage and pathological substance delivery (Zhang et al., 2018). Dysregulation of endothelial function plays a crucial role in the early stages of many human diseases. Studies have manifested that patients with early endothelial dysfunction are more susceptible to cardiovascular and AMD in later life (Cheung and Wong, 2014). However, the mechanism of choroidal vascular endothelial dysfunction in CNV is still elusive. Furthermore, the limitations of anti-VEGF agents in the treatment of CNV, such as new vessels reappearance and decreased sensibility to the ophthalmic preparations after injection, indicate that the identity of novel regulatory mechanisms other than the classic VEGF signaling pathway axis requires clarification (Huang et al., 2016; Yeo et al., 2019). In recent years, the role of lncRNAs in various diseases, such as diabetes, obesity, cardiovascular diseases, and nervous system diseases, has drawn an increasing interest. We also investigated the role of lncRNA PNKY in endothelial cells. PNKY was downregulated in RF/6A cells upon hypoxic stress. PNKY knockdown promoted the RF/6A cell proliferation, migration, and tube formation, protecting cell against apoptosis from hypoxia stress. In contrast, PNKY overexpression suppressed RF/6A cells vasogenic function and aggravated the cell apoptosis. These results suggested that PNKY could regulate RF/6A cell function *in vitro*.

LncRNAs mainly exert biological functions through the following four mechanisms: act as a guide for specific proteins and promote their localization within cells or to specific genetic loci, bind to and inhibit a protein target or titrate out protein or other non-coding RNA, serve as a central platform for recruiting multiple proteins into the ribonucleoprotein complex, and act as a cellular signal (Connerty et al., 2020). The expression of many lncRNAs is associated with neighboring genes, and they affect the expression of adjacent genes by interacting with RNA-binding proteins; their functions can therefore be inferred from the co-expression of interacting genes (Zhang et al., 2017). It is reported that PNKY interacts with PTBP1, a heterogeneous nuclear ribonucleoprotein and a classical multifunctional RNA binding protein that binds to downstream targets through specific sequences and regulates alternative splicing and mRNA decay. PTBP1 is engaged in many biological processes, including maintenance of cell structure and motility, immunity, protein metabolism, and cell cycle (Shan et al., 2018). Our results showed that PTBP1 was bound to PNKY, and its silencing could inhibit the regulatory function of PNKY in RF/6A cells, indicating that PNKY regulates the function of RF/6A cells through combining to PTBP1.

We further investigated the mechanisms of PNKY/PTBP1-mediated angiogenic function. We performed the TargetScan database and found that miR-124 was the target gene of PTBP1. The role of miR-124 in human diseases has also been widely demonstrated, Bao et al. (2020) and Jin et al. (2020) found the antiapoptotic effect of miR-124. However, its mechanism in cell proliferation, apoptosis, and angiogenesis remains unclear. In our study, we found that miR-124 was at a higher level in laser induced CNV model than that in the control. PTBP1 silencing could promote the level of miR-124 in RF/6A cells, and PNKY overexpression downregulated the expression of miR-124. To confirm the interaction among the three *in vitro*, we conducted the loss-of-function and gain-of-function experiments. As shown, miR-124 mimic promoted the viability, proliferation, migration, and tube formation activity of RF/6A cells, while PNKY overexpression could block the function of miR-124. When PTBP1 was silenced, PNKY was then unable to exert this inhibitory effect. These results indicated that PNKY/PTBP1 plays antiangiogenic function by regulating the expression of miR-124.

Also, we exploited the starBase tool to further predict the targets of miR-124 for a deeper insight into the regulatory pattern of miR-124. We found a target gene of miR-124, BIM (Bcl-2-like 11), and a BCL2 cell death interaction mediator, which is a prominent pro-apoptotic BCL2 family member. BIM could induce apoptosis either by counteracting pro-survival members of families or by directly binding to active pro-apoptotic effectors (Malishev et al., 2021). Our results showed that the expression of BIM was decreased by miR-124 and increased by PNKY overexpression. When PTBP1 was knocked down, the promotion effect of PNKY on BIM was then weakened. These results mentioned above suggested that PNKY/PTBP1–miR-124-BIM network is involved in the development of CNV.

The role of lncRNAs as regulators has been widely studied, while little is known about the origin. Several mechanisms might be involved in the regulation of the lncRNA expression level. Chromatin state is one of the most significant mechanisms that directly regulate differential lncRNAs expression profiles of cell types, such as the interaction between DNA methylation and histone modification. The key transcription factors of protein-coding genes and some miRNAs can also regulate the expression of lncRNAs. In addition, it was found that a small fraction of lncRNA (pancRNAs) can be expressed by bidirectional promoters (Wu et al., 2014). In our study, we demonstrated that lncRNA PNKY regulate the expression of key genes through various molecular mechanisms to influence biological processes. How lncRNA PNKY is reduced in CNV diseases is worthy of further study.

In conclusion, we utilized the endothelial cells and laser-induced CNV mice to investigate the role of PNKY in the development of CNV. PNKY silencing promoted endothelial angiogenic effects *in vitro* and deteriorated pathological angiogenesis *in vivo*. PNKY/PTBP1-miR-124-BIM network was involved in the development of CNV. This study sheds new light on a new pathogenesis for the development of CNV and provides a considerable target for the treatment of ocular neovascularization diseases.

## Data Availability

The original contributions presented in the study are included in the article/Supplementary Material; further inquiries can be directed to the corresponding authors.
