# Supplementary material for: Long Non-Coding RNA PNKY Modulates the Development of Choroidal Neovascularization
Source: Front Cell Dev Biol. 2022 Feb 21;10:836031. doi: 10.3389/fcell.2022.836031 (PMC8899849; doi:10.3389/fcell.2022.836031)
Supplement: Supplementary file 1 [file Table1.DOCX]

Supplementary Material

## Supplementary Figures


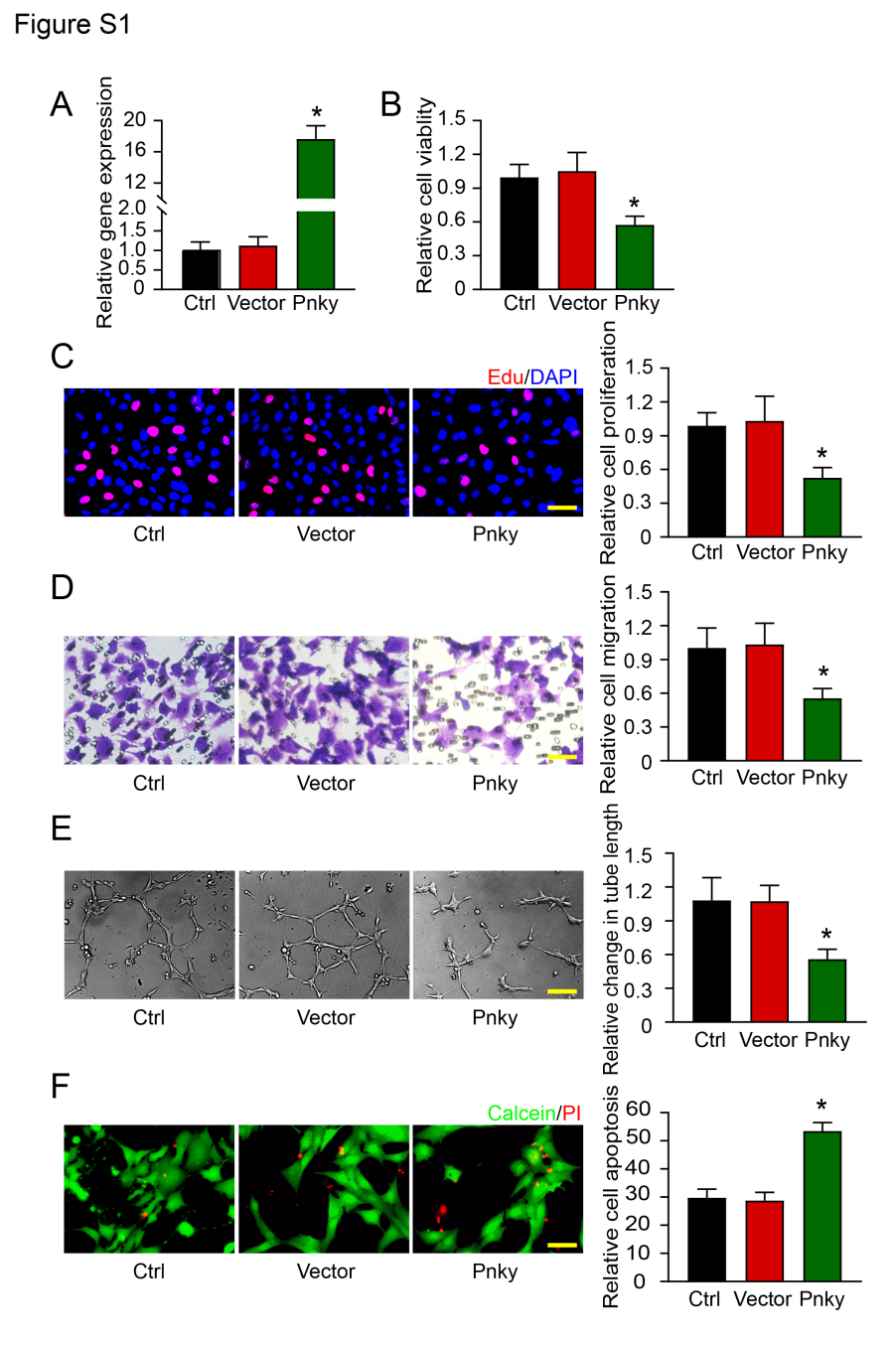


**Supplementary Figure 1. lncRNA-Pnky overexpression decreases endothelial angiogenic function *in vitro.*** RF/6A cells were transfected with pcDNA3.0 vector (Vector), pcDNA3.0-Pnky (Pnky), or left untreated (Ctrl) for 24 h before further processed. **(A)** qRT-PCRs were performed to detect Pnky expression (n=4). **(B)** Cell viability was detected using MTT method (n=4). **(C)** Cell proliferation was detected using EdU detection kit. Blue: DAPI; red: EdU. (Scale bar, 50 μm, n=4). **(D)** Migration of RF/6A cells was measured using transwell assay and quantification of migrated cells was performed (Scale bar, 50 μm, n=4). (E) The tube-like structures were observed 4 h after cells seeding on the matrix. Average length of the capillary-like tubular structures for each field was statistically analyzed (Scale bar, 200 μm, n=4). All data were from at least three independent experiments. All significant difference was determined by one-way ANOVA followed by Bonferroni’s post-hoc test. *P* < 0.05 versus Scr siRNA.


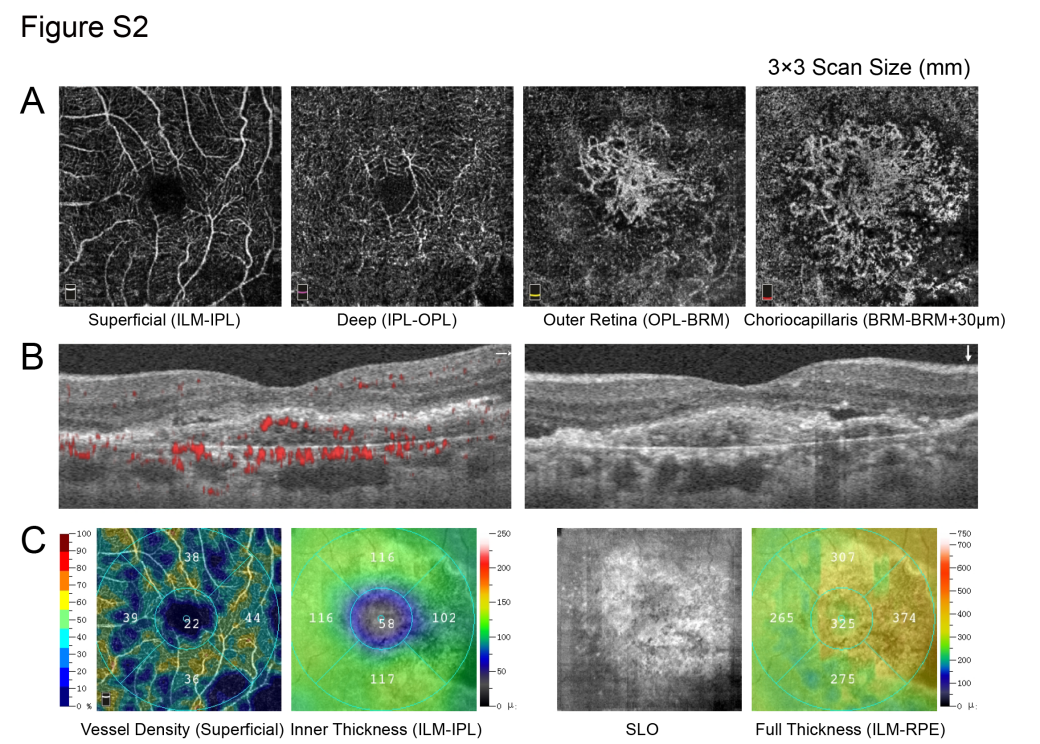


**Supplementary Figure 2. Choroidal neovascularization diagnosed by OCTA.** The CNV network was observed by optical coherence tomography angiography (OCTA). **(A)** Different levels of retinal vessels and the CNV lesion in outer retina and choriocapillaris layers (3×3 Scan Size (mm)). **(B)** Transverse and sagittal views of the CNV lesion site. **(C)** Vessel density and tissue thickness in different parts of the retina.
